# Collective responses in electrical activities of neurons under field coupling

**DOI:** 10.1038/s41598-018-19858-1

**Published:** 2018-01-22

**Authors:** Ying Xu, Ya Jia, Jun Ma, Tasawar Hayat, Ahmed Alsaedi

**Affiliations:** 1 Institute of Biophysics and Department of Physics, Central China Normal University, Wuhan, 430079 China; 2 Department of Physics, Lanzhou University of Technology, Lanzhou, 730050 China; 3 Department of Mathematics, Faculty of Science, King Abdulaziz University, Jeddah, 21589 Saudi Arabia; 4 Department of Mathematics, Quaid-I-Azam University 45320, Islamabad, 44000 Pakistan

## Abstract

Synapse coupling can benefit signal exchange between neurons and information encoding for neurons, and the collective behaviors such as synchronization and pattern selection in neuronal network are often discussed under chemical or electric synapse coupling. Electromagnetic induction is considered at molecular level when ion currents flow across the membrane and the ion concentration is fluctuated. Magnetic flux describes the effect of time-varying electromagnetic field, and memristor bridges the membrane potential and magnetic flux according to the dimensionalization requirement. Indeed, field coupling can contribute to the signal exchange between neurons by triggering superposition of electric field when synapse coupling is not available. A chain network is designed to investigate the modulation of field coupling on the collective behaviors in neuronal network connected by electric synapse between adjacent neurons. In the chain network, the contribution of field coupling from each neuron is described by introducing appropriate weight dependent on the position distance between two neurons. Statistical factor of synchronization is calculated by changing the external stimulus and weight of field coupling. It is found that the synchronization degree is dependent on the coupling intensity and weight, the synchronization, pattern selection of network connected with gap junction can be modulated by field coupling.

## Introduction

Neurons are the basic structural and functional units of nervous system. The information processing and encoding are approached by exchange of neurotransmitter via chemical and electrical action, and the signal receiving is dependent on synapses between neurons^1–7^. For example, *Qin et al*.^5^ discussed the biological function of autapse connection to neurons and confirmed that autapse driving can enhance self-adaption of neurons. The biological nervous system is usually very complex and hierarchical, and it contains very complex inner structures, such as multiple subsystems. Therefore, the research in internal competition and cooperation behavior of complex system has to resort to the sampling and system analysis. The related studies have very great significance on understanding the evolution of collective behaviors of complex systems, and it is also helpful to predict the dynamical behavior of control systems. For an isolated neuron, geometry and anatomical structure often account for biological function, for example, autapse formation could be associated with injury in axon thus auxiliary loops are developed to propagate the blocked signals^8^. Moreover, the impact of different topology networks in various neuron models has been widely investigated by using bifurcation analysis. Indeed, coupling strength^9,10^, time delay^11^, spatial in homogeneity stimulus^12^, and noise^13,14^ can affect the synchronization and stability of network. The network is composed of many nodes, the statistical analysis based on mean field theory and nonlinear analysis from sampled time series of observable variables are often used to detect the synchronization stability and pattern selection^15,16^. However, it could be difficult for generic sampling and statistical analysis when network is composed of many sub-networks, but pattern selection could be effective to understand the transition in collective behaviors. On the other hand, the use of speckle graph dynamics is much helpful to study the group discharge behaviors of neuronal networks, thus the spiral wave characteristics of spatial distribution of electrical activity in the cerebral cortex^17^ can be understood. The spiral wave is self-sustained, and the developed spiral waves in cortex can change the collective behaviors in electrical activities as a stable pacemaker. The potential mechanism for emergence of spiral wave and its dynamics are discussed in ref.^18^, which local and diffusive poisoning in ion channels^19–21^ can generate defects to trigger continuous waves and further occurrence of spiral waves after symmetrical breaking^22^ in the profile. For example, *Hu et al*.^22^ confirmed that symmetry breaking of target wave in the media accounts for emergence of multiarmed spiral wave, which can be developed from a group of spiral waves with single arm under appropriate condition, thus the potential formation mechanism of multiarmed spiral wave in the media is explained. *Gong et al*^23^. discussed the influence of time delay and channel blocking on multiple coherence resonance in network composed of Hodgkin-Huxley neurons. *Qin et al*.^24^ explained the defect mechanism for autapse driving in network by using negative feedback and the wave propagation was also discussed. *Xiao et al*.^25^ reported the dynamics in spatiotemporal patterns in network by changing the excitability. *Xu et al*.^26^ explained the new mechanism of spatial coherence resonance by imposing noise and periodical pacing in different local areas in the network, and it is found that local forcing-induced wave can be broken in the area driven by noise and spiral waves are formed under appropriate noise intensity in the network. *Li et al*.^27^ discussed the selection and breakup of spiral wave in a coupled network driven by Gaussian colored noise. *Qian et al*.^28^ analyzed all oscillatory complex networks consisting of non-oscillatory nodes by using the dominant phase-advanced driving method, the oscillation sources and wave propagation paths of the self-sustained target waves in excitable small-world networks were explored explicitly.

In fact, spatial patterns such as spiral wave, target wave and Turing patterns can also be found in Reaction-diffusion systems and neuronal networks as well. Pattern formation in nonlinear complex systems is one of the central problems of the natural, social, and technological sciences. Since Turing firstly proposed reaction-diffusion (RD) theory to model the range of spatial patterns observed in the developing embryo^29^, RD models have been studied extensively to explain patterns in both epidemiology and ecosystems^30^. It is found that cross diffusion^31^, time delay^32,33^ and functional response^34^ can induce the appearance of stationary patterns. It is confirmed that the standard multiple-scale analysis and exact Turing regions can be obtained^35^ to develop kinds of spatial patterns. In fact, spiral wave, target wave or patched invasion can be observed^36,37^ when the diffusion coefficients are the same (Turing theory does not hold). Based on the data observations, it is revealed that multiple scale spatial patterns^38^, isolation degree^39^ and spatial scaling laws^40^ play important roles on the resilience of the biological systems and thus they have implications on population protection or diseases control. In cardiac tissue and also cortex, the emergence and survival of spiral waves, continuous pulses could disturb signal propagation and information processing, thus normal electrical activities in cortex and wave propagation in cardiac tissue can be perturbed for inducing possible diseases. Therefore, it is important to apply effective schemes^40,41^ to remove and suppress spiral waves. Based on the driven-synchronization, the rotating electric field (REF) can be utilized to “smooth” a heterogeneous medium and suppress turbulence^42^. *Chen et al*.^43,44^ found that the REF can induce wave emission from heterogeneity in excitable media, which may provide a potential application to utilized the existed defects on cardiac tissue as the second sources to remove pinned spiral waves^45^.

That is, the effect of electric field on cardiac tissue can often be described by adding gradient terms in the membrane potential of media described by the Reaction-Diffusion system. In the cortex and neuronal network, it is a challenge to deal with this problem that the effect of electromagnetic induction can be understood from physical view. The influence of the biological effects of electromagnetic fields on human health has been investigated, and electromagnetic radiation-induced disease and abnormality in nervous systems (such as memory loss and other symptoms) were also discussed^46–55^. On the other hand, the dynamics of neuronal activities in presence of electromagnetic field also became attractive^56–58^. In fact, by further improving the computational models for neuron^59,60^ and cardiac tissue^61,62^, the physical effect can be described by using magnetic flux associated with time-varying electromagnetic field. For example, *Lv et al*.^63^ proposed a four-variable neuron model developed from the previous Hindmarsh-Rose neuron model^59,60^ by adding the variable for magnetic flow, which memristor is also used to bridge the coupling between membrane potential and magnetic flux^63^. Inspired by this model, the transition of electrical activities in neurons induced by electromagnetic radiation has been investigated extensively^64–68^, particularly, the same scheme was carried out on cardiac tissue, and two death mechanisms^61,62^ in heart induced by electromagnetic radiation are explained. For a further survey and guidance, readers can find clues in refs^69,70^ and references therein. In most of the previous works, synapse connection and gap junction are regarded as the main bridge to benefit signal exchange and wave propagation between neurons. In this paper, we argued that field coupling could be another effective way for signal propagation in network because field coupling can realize phase synchronization between neurons^71^. The potential mechanism is explained that each neuron can contribute to the distribution of electric field, and the electrical activity of each neuron will be affected by other neurons by triggering different electric fields in the network.

## Model and scheme

The Hindmarsh-rose neuron model^59,60^ mainly highlights the main nonlinear dynamic characteristics of mollusk neurons, the dynamical kinetics can be approached by the ordinary differential equations (ODEs) composed of three variables shown in ref.^59^. As proposed and discussed in refs^63,64^, magnetic flux is used to describe the effect of electromagnetic induction^72^, and memristor is considered to realize coupling between membrane potential and magnetic flux thus the induction field and action potential can be bridged in physical view. The dynamical neuron model is described by

1 $$\{\begin{array}{l}\frac{dx}{dt}=y-a{x}^{3}+b{x}^{2}-z+I-k\rho (\phi )x\\ \frac{dy}{dt}=c-d{x}^{2}-y\\ \frac{dz}{dt}=r[s(x+1.56)-z]\\ \frac{d\phi }{dt}={k}_{1}x-{k}_{2}\phi \end{array}$$

where the variable *x*, *y*, *z* represents the membrane potential, slow current for recovery variable, and adaption current, respectively. The variable *I* defines the external forcing current, and the fourth variable *φ* describes the magnetic flux across membrane. The *ρ*(*φ*) is the memory-conductance of a magnetic flux-controlled memristor and it is approached as *ρ*(φ) = *α* + 3*β*φ^2^, *kρ*(*φ*)*x* denotes induction current and *k* is induction coefficient. Further detailed description for parameters can be found in refs^63,64^. The induction current is calculated as follows

2 $$i^{\prime} =\frac{dq(\phi )}{dt}=\frac{dq(\phi )}{d\phi }\frac{d\phi }{dt}=\rho (\phi )\frac{d\phi }{dt}=-k\rho (\phi )x$$

That is, the effect of electromagnetic induction and change of electric field is described by induction current. The memductance of memristor is dependent on the magnetic flux and time-varying induction current is imposed on the neuron. The memristor^73^ is often used as nonlinear electric device in setting nonlinear circuits and synchronization can be approached by using memristor coupling^74,75^. It is also effective to describe the effect of electromagnetic induction in neuronal activities. From physical view, each neuron and cell can be thought as an non-uniform charged body because the distribution of charged ions are not distributed in uniform way, and thus complex field distribution can be triggered in intracellular and extracellular space. The effect of electric field could be distinct and the time-varying electromagnetic field plays important role in changing the exchange of charged ions. In biological and nervous systems, contribution to the field distribution from each neuron could be different and shows diversity, therefore, appropriate weight is considered in the neuronal network. For simplicity, in a chain-like network, the position of neuron is marked with subscript “*i*”, another neuron is marked with subscript “*j*”, it is supposed that the contribution to field intensity is inversely proportional to the number error as *Γ*_*ij*_ = *W*/│*i* − *j*│, and *W* is the weight value contributed to the field intensity. As a result, the network can be approached by gap junction coupling and field coupling; the dynamical equations are described by

3 $$\{\begin{array}{l}\frac{d{x}_{i}}{dt}={y}_{i}-a{{x}_{i}}^{3}+b{{x}_{i}}^{2}-{z}_{i}+I+D({x}_{i+1}+{x}_{i-1}-2{x}_{i})-k\rho ({\phi }_{i}){x}_{i}\\ \frac{d{y}_{i}}{dt}=c-d{{x}_{i}}^{2}-{y}_{i}\\ \frac{d{z}_{i}}{dt}=r[s({x}_{i}+1.56)-{z}_{i}]\\ \frac{d{\phi }_{i}}{dt}={k}_{1}{x}_{i}-{k}_{2}{\phi }_{i}+{D}_{0}({\phi }_{i}-\sum _{\begin{array}{c}j=1\\ i\ne j\end{array}}\frac{W}{|i-j|}{\phi }_{j})\end{array}$$

**Figure 1:**
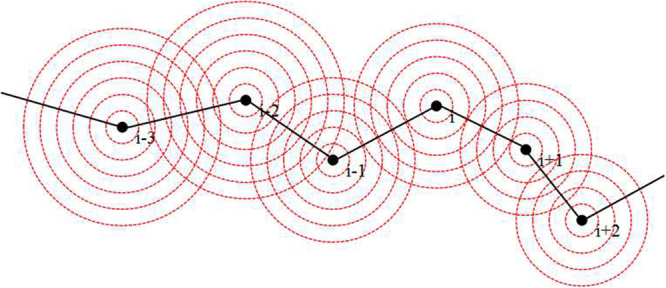
Schematic diagram of field coupling between neurons, the red circles mark the distribution of electric field. The black point represents the neuron, black line means connection between neuron, the red circles denotes the propagation of electric field and the same circle means the same intensity distribution for electric field. According the Coulomb’s law in physics, the intensity of electric field can be described by *E* = *Q*/4πε*r*^2^, where *Q* means the charge value, *ε* is the dielectric coefficient; *r* denotes the distance from charged cell to field point (detection position).

The subscript *i* indicates the position of the neuron in the network, *D* is the coupling coefficient between adjacent neurons via electric synapse coupling, *D*_0_ describes the field interaction between neurons. *W* represents the intensity of the field effect associated with distance between neurons. According to Eq. (3), increasing the value for intensity of field coupling, positive feedback will enhance the electromagnetic induction effect, and stronger induction current will be induced to decrease the excitability of neurons. The parameters are selected as *a* = 1.0, *b* = 3.0, *c* = 1.0, *d* = 5.0, *s* = 4.0, *α* = 0.1, *β* = 0.02, *k* = 0.9, *k*_1_ = 0.4, *k*_2_ = 0.5. The field coupling is illustrated in Fig. 1.

To discern the collective behavior of neurons, a statistical factor of synchronization^24,26,69^ is calculated to find synchronization approach based on mean field theory, and it reads as follows

4 $$F=\frac{{\bf{1}}}{N}\sum _{i=1}^{N}{x}_{i},\quad R=\frac{ < \,{F}^{2} > - < F{ > }^{2}}{\frac{{\bf{1}}}{N}\sum _{i=1}^{N}[ < {x}_{i}^{2} > - < {x}_{i}{ > }^{2}]}$$

where *N* is the node number of neuronal network, < * > describes the average of variable over time, for simplicity, a transient period *T* = 2000 time units will be used for numerical studies. Perfect synchronization is approached when *R* is close to 1, and non-perfect synchronization is detected together with emergence of appropriate pattern distribution when *R* is close to zero.

## Numerical results and discussion

**Figure 2:**
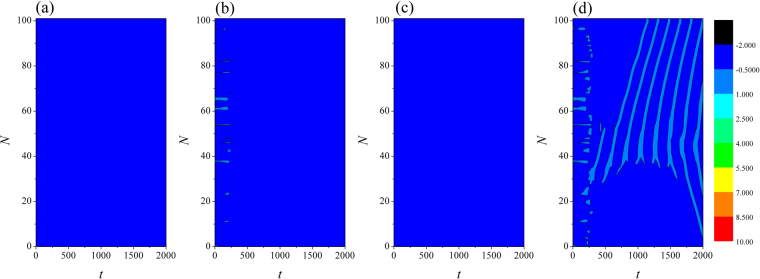
Developed spatial patterns of network and sampled time series are calculated when network is modulated by field coupling. Coupling intensity between nodes *D* = 0.5, *W* = 1.0, for (**a**) *D*_0_ = 0.0001, *I* = 1.0; (**b**) *D*_0_ = 0.00048, *I* = 1.0; (**c**) *D*_0_ = 0.0001, *I* = 1.2; (**d**) *D*_0_ = 0.00048, *I* = 1.2. The snapshots are shown in color scale.

**Figure 3:**
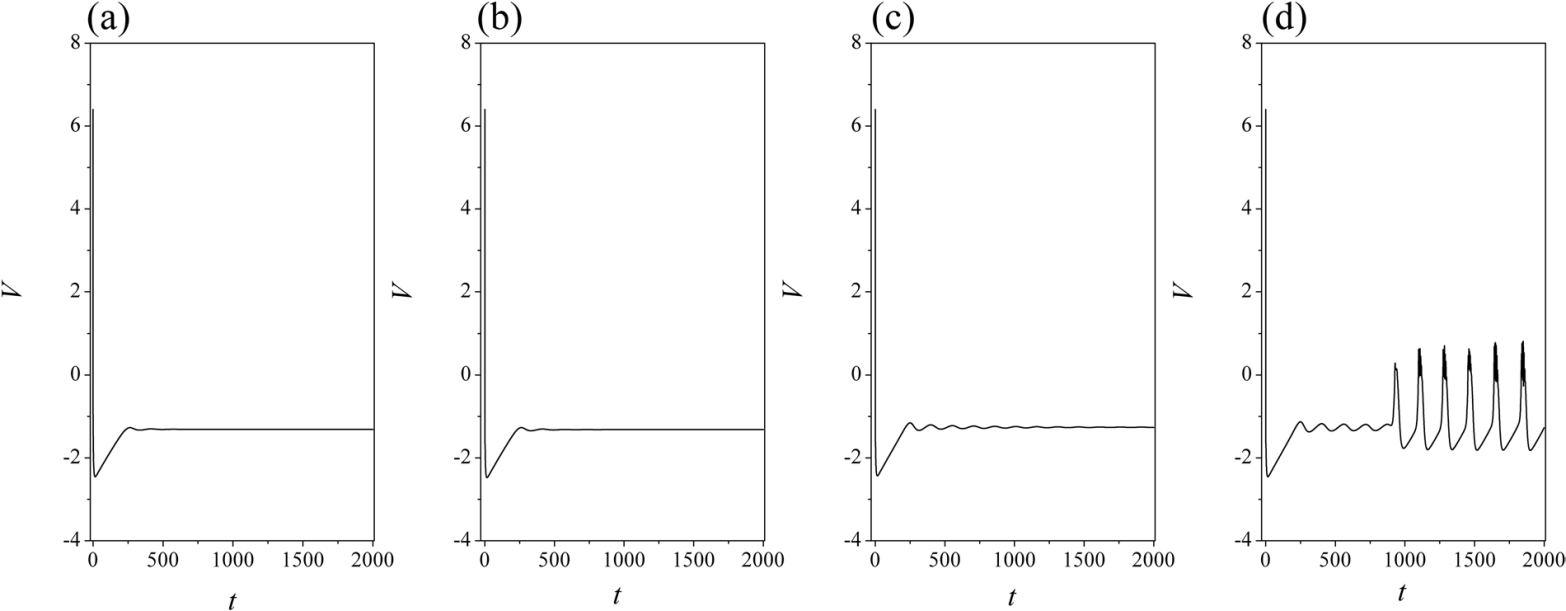
Sampled time series for membrane potential from node (*i* = 80) are calculated. Coupling intensity between nodes *D* = 0.5, *W* = 1.0, for (**a**) *D*_0_ = 0.0001, *I* = 1.0; (**b**) *D*_0_ = 0.00048, *I* = 1.0; (**c**) *D*_0_ = 0.0001, *I* = 1.2; (**d**) *D*_0_ = 0.00048, *I* = 1.2.

In the numerical section, the fourth order Runge-Kutta algorithm is used to approach solutions for the dynamical equations. The chain network is composed of 100 neurons, time step is set as *h* = 0.01. The initial values are selected random values as (0.3 + *ξ*, 0.1 + *ξ*, 5 + *ξ*, 0, 0.2 + *ξ*), where *ξ* is a random number ranged from 0 to 1. Transient period for calculation is about 2000 time units, coupling coefficient *D* = 0.5. In order to illustrate the effect of field superposition on neuronal discharges, different external stimuli *I* are applied when the chain neuron *W* is set to 1. The spatiotemporal evolution of membrane potentials are calculated for dynamical analysis when the node position driven by external stimulus current is changed. Furthermore, the distribution for factors of synchronization on the network is calculated by selecting different external stimuli *I* and *D*_0._ At first, the weight of electric field contribution is fixed at *W* = 1, the intensity of field coupling is adjusted to observe the development of spatial patterns(spatial distribution of membrane potential), the results are plotted in Fig. 2 and sampled time series for membrane potential of neuron connected to node (*i* = 80) are shown in Fig. 3.

The excitability of neuron mainly depends on the external forcing current, and larger external stimuli are much helpful to excite neurons and the collective behaviors of network will present oscillating state. When the subthreshold forcing is applied on the network, diversity in initial settings can trigger short transient oscillation and then the network become quiescent state occupied the network completely. By further increasing the intensity of external stimuli, the excitability of neuron is also enhanced, the quiescent neurons are waken up to present spiking and bursting states. Indeed, the field coupling contributes magnetic flux and induction current, as a result, the mode in electrical activities are controlled. As shown in Figs 2d, 3d, further increasing the intensity of field coupling, diversity in magnetic flux and induction currents are induced, as a result, each neuron shows different excitability to trigger appropriate oscillating behaviors in electrical activities.

**Figure 4:**
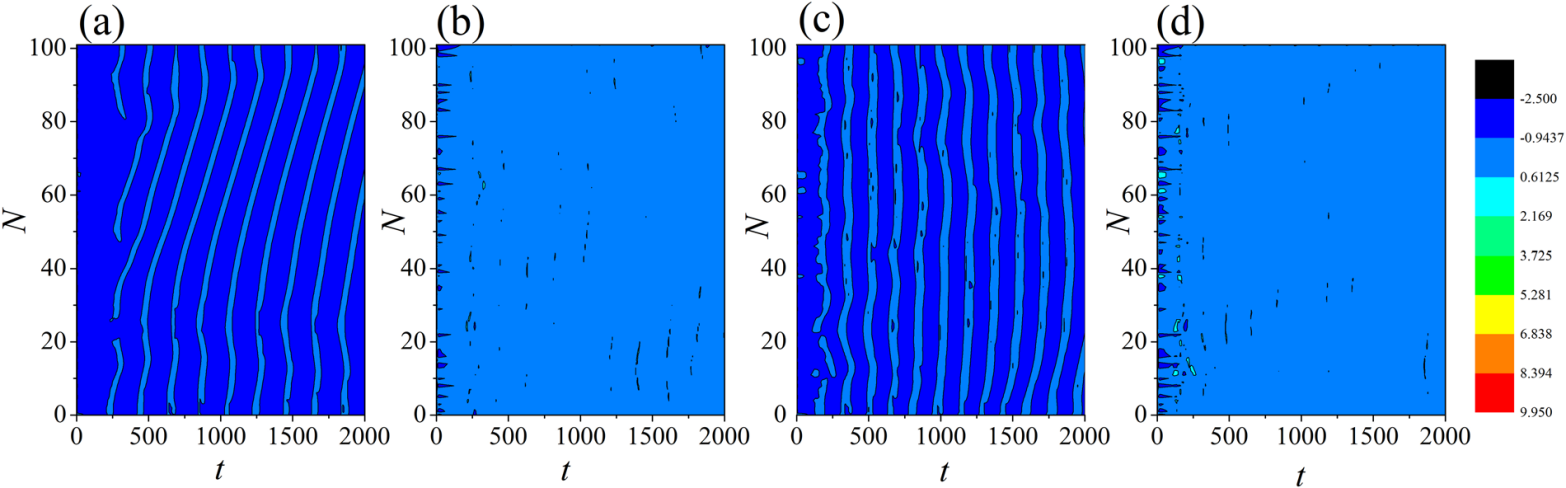
Evolution of spatial patterns is calculated at *W* = 1, *D* = 0.5, the transient period for calculating is 2000 time units. For (**a**) *D*_0_ = 0.0001, *I* = 1.3; (**b**) *D*_0_ = 0.001, *I* = 1.3; (**c**) *D*_0_ = 2.0, *I* = 0.0001; (**d**) *D*_0_ = 2.0, *I* = 0.001.

**Figure 5:**
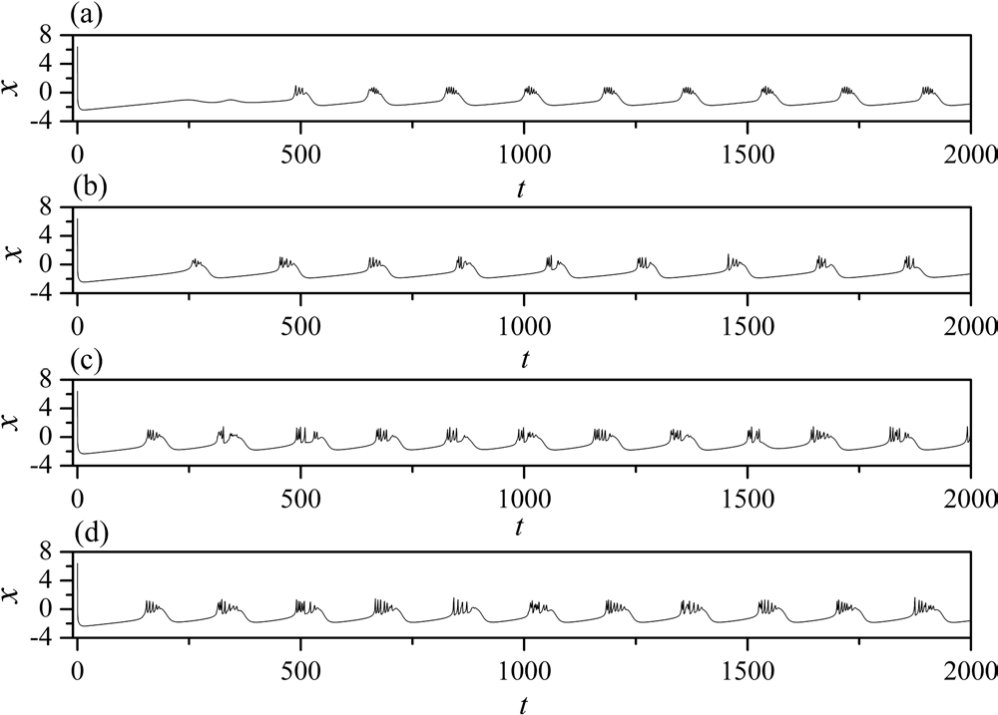
Sampled time series for membrane potential of neuron (*i* = 80) are calculated by applying different stimulus current, at *W* = 1, *D* = 0.5. For (**a**) *D*_0_ = 0.0001, *I* = 1.3; (**b**) *D*_0_ = 0.001, I = 1.3; (**c**) *D*_0_ = 2.0, *I* = 0.0001; (**d**) *D*_0_ = 2.0, *I* = 0.001.

The results in Fig. 4 confirmed that the field coupling plays important role in regulating the collective behaviors of the network, while slight difference in external stimuli can also enhance the developed state when the intensity of field coupling is highly increased. The sampled time series for some nodes found that bursting behaviors are enhanced under field coupling even stimuli is much weak, the results are shown in Fig. 5_._

**Figure 6:**
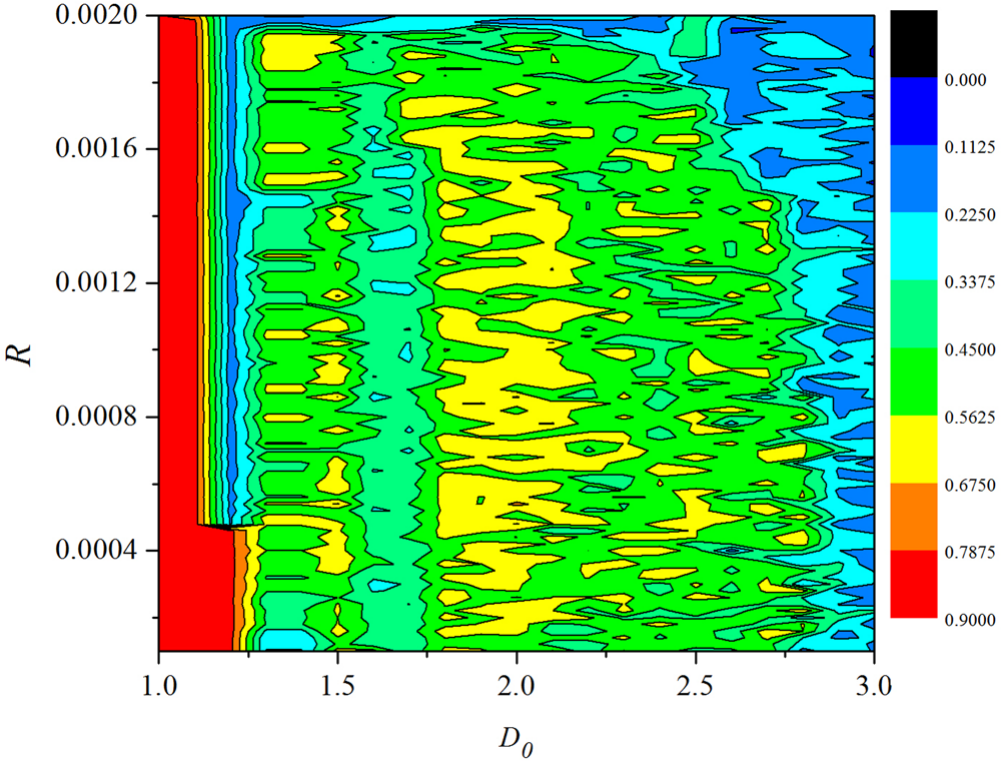
Distribution for synchronization factors is calculated by changing the intensity of field coupling and external stimulus. The snapshots are plotted in color scale.

The change in the discharge period of the neuron is caused by the change of the field interaction between each neuron and the other neurons, that is, *D*_0_ is positively correlated with the neuronal discharge period *T*. It is found that the bursting states show distinct difference and the response intervals are much different. The field coupling can drive all neurons to give appropriate response in time. The distribution for factors of synchronization is calculated in the two-parameter space, and the contribution from field coupling and external forcing is estimated, the results are shown in Fig. 6.

**Figure 7:**
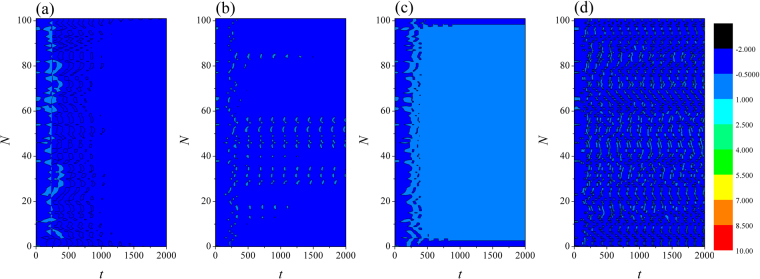
Developed spatial pattern in the network. The weight coefficient *W* = 1 and electric coupling *D* = 0, for (**a**) *I* = 1.2, *D*_0_ = 0.0001; (**b**) *I* = 1.2, *D*_0_ = 0.00048; (**c**) *I* = 1.0, *D*_0_ = 0.0001; (**d**) *I* = 2.0, *D*_0_ = 0.0001.

**Figure 8:**
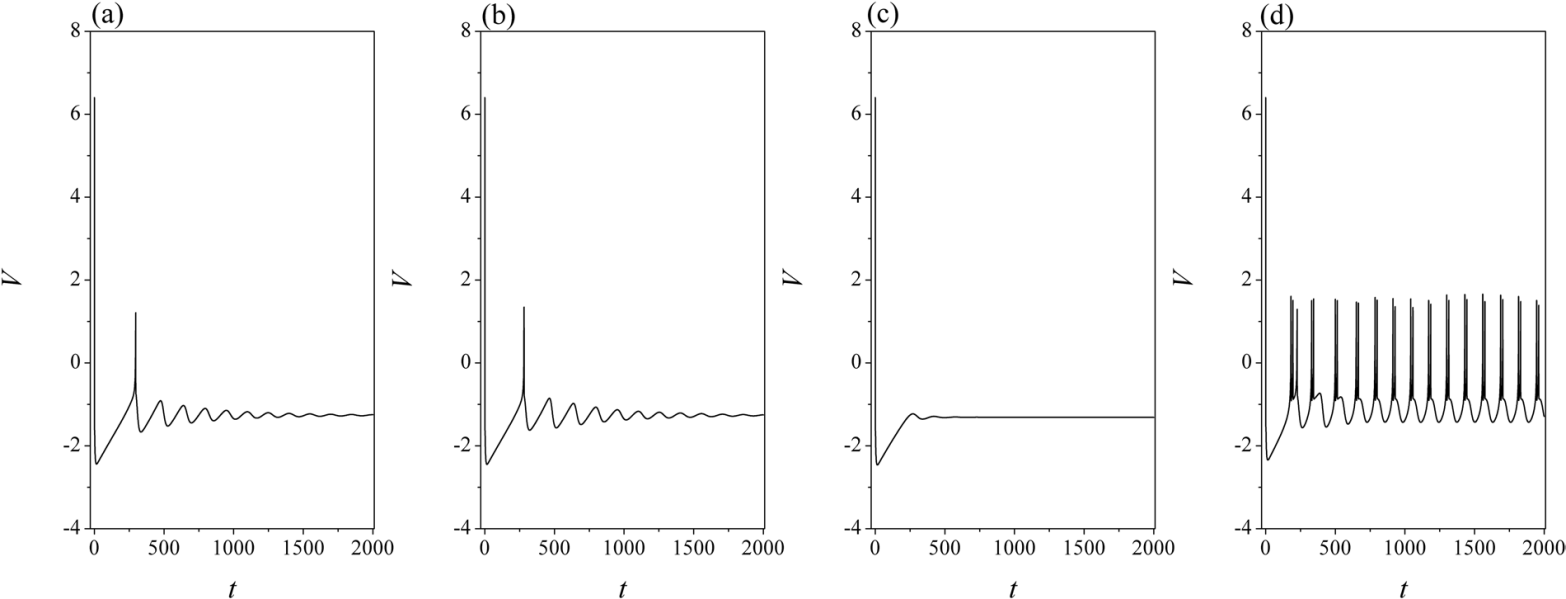
Sampled time series for membrane potential of neuron (*i* = 80) are calculated by applying different stimulus current. The weight coefficient *W* = 1 and electric coupling *D* = 0, for (**a**) *I* = 1.2, *D*_0_ = 0.0001; (**b**) *I* = 1.2, *D*_0_ = 0.00048; (**c**) *I* = 1.0, *D*_0_ = 0.0001; (**d**) *I* = 2.0, *D*_0_ = 0.0001.

**Figure 9:**
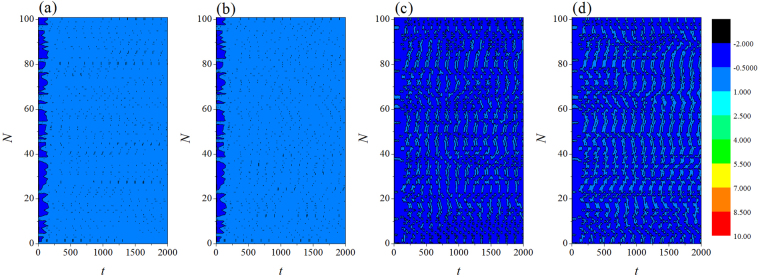
Developed spatial pattern in the network. The weight coefficient *W* = 1 and electric coupling *D* = 0, *I* = 2.0, for (**a**) *D*_0_ = 0.0; (**b**) *D*_0_ = 0.00001; (**c**) *D*_0_ = 0.0001; (**d**) *D*_0_ = 0.0002.

**Figure 10:**
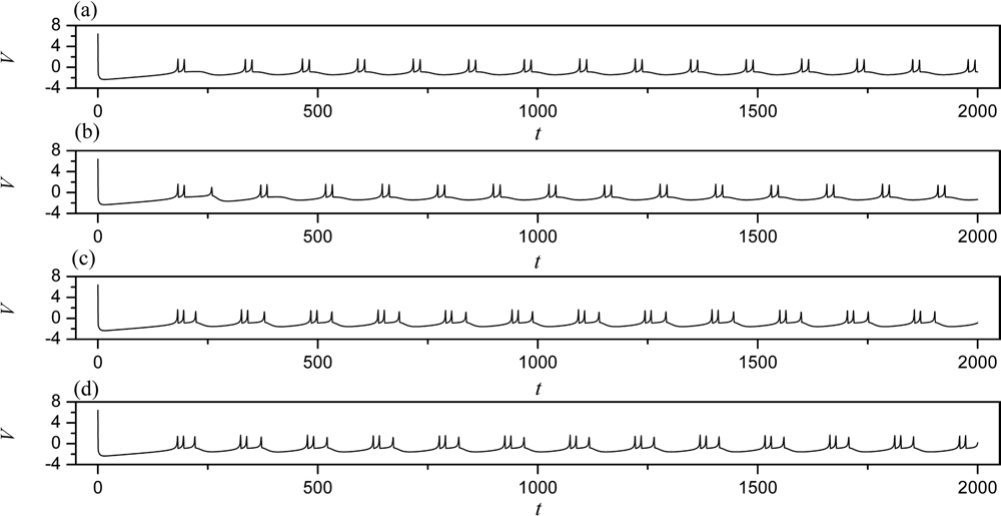
Sampled time series for membrane potential of neuron (*i* = 80) are calculated by applying different intensities of field coupling. The weight coefficient *W* = 1 and electric coupling *D* = 0, *I* = 2.0, for (**a**) *D*_0_ = 0.0; (**b**) *D*_0_ = 0.00001; (**c**) *D*_0_ = 0.0001; (**d**) *D*_0_ = 0.0002.

It is confirmed that the synchronization degree is high when external forcing current is weak that excitability is low, as a result, the effect of diversity in initials setting can be suppressed by field coupling. To discern the effect of field coupling, the intensity of electric coupling is removed as *D* = 0, then external forcing current and field coupling are considered, the results are shown in Figs 7, 8, 9, 10.

It is confirmed that weak excitability can’t support continuous oscillating, while field coupling can enhance the oscillating behaviors in electrical activities. The external stimuli mainly contribute the electrical activities when the intensity of field coupling is weak. Furthermore, the field coupling is enhanced to observe the evolution of collective behaviors, the results are shown in Figs 9, 10.

**Figure 11:**
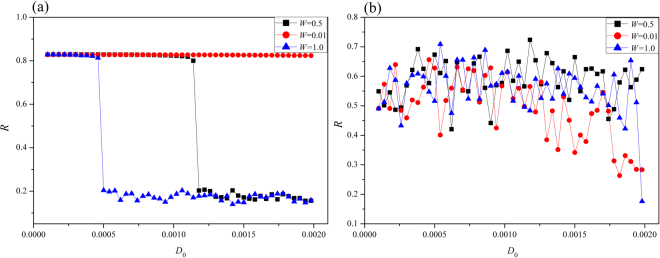
Dependence of synchronization factor on weight *W* and intensity of field coupling is calculated. The external stimulus is set for (**a**) *I* = 1.2, (**b**) *I* = 2.0.

In the case of high excitability by applying stronger external stimuli, the oscillating behaviors and bursting states can be increased greatly under field coupling. As shown in Fig. 10, multiple modes in electrical activities are induced by further increasing the intensity of field coupling, and all the neurons can give rapid response under field coupling. Extensive numerical results confirmed synchronization can be approached when electric coupling is set as *D* = 0.5 when diversity in initials setting is removed. As a result, the developed states show some difference when field coupling is triggered in different transient periods. Furthermore, the effect of weight contributed to the supimposed field is discussed, the transition of synchronization factors is estimated by changing the intensity of field coupling, the results are plotted in Fig. 11.

It is found that the weight in field coupling also contributes to the synchronization approach of the network. When the weight for field coupling is much small, the synchronization factors show slight difference and synchronization mainly depends on the electric coupling. The role of weight becomes most important when field coupling is increased in intensity because the contribution from each neuron becomes distinct different than other neurons. When the external stimulus is decreased to *I* = 1.2, the field coupling can modulate the synchronization behavior greatly (decreases the synchronization) with increasing the intensity of field coupling. However, in case of high excitability and stronger stimulus, the oscillating behaviors and evolution of network mainly depend on the stimuli and the effect of weight shows slight difference on synchronization approach.

In a summary, the evolution of collective behaviors and synchronization degree are dependent on the electric coupling via gap junction, intensity of field coupling, weight for superimposed field, diversity in initials setting. Due to the memory effect of memristor associated with magnetic flux and effect of initials diversity becomes important and distinct. The external forcing current changes the excitability of neuron and the electrical activities of neuronal network can be changed directly. Field coupling can modulate the collective behaviors of network by inducing induction currents with diversity and diversity in excitability of network is triggered. The development and evolution of collective electrical activities of neuronal network are contributed by field coupling, electric coupling when external forcing is fixed. On the other hand, the field coupling between neurons can be considered as external electromagnetic radiation on isolated neuron. Readers can extend this study by setting other networks with different connection types when field coupling is considered.

## Conclusions

An improved neuron model, which the membrane potential and magnetic flux are bridged by using memristor, is used to describe the local kinetics of chain network of neurons. The effect of electromagnetic field is described by magnetic flux. Each neuron is regarded as an charged body and is controlled by the electric field triggered by other neurons. A weight value is introduced to discern the contribution to superimposed field from each neuron, field coupling between neurons is described by exchange of magnetic flux. It is found that field coupling between neurons can change the magnetic flux and induction current, and then the excitability of neurons are changed to modulate the collective behaviors of electrical activities in neuronal network. It could give instructive clues to understand the signal encoding and exchange when synapse coupling is suppressed.
